# Dose Analysis of Photobiomodulation Therapy in Stomatology

**DOI:** 10.1155/2020/8145616

**Published:** 2020-09-16

**Authors:** Chuan-Tsung Su, Chung-Ming Chen, Chun-Cheng Chen, Jih-Huah Wu

**Affiliations:** ^1^Institute of Biomedical Engineering, College of Medicine and College of Engineering, National Taiwan University, Taipei 10617, Taiwan; ^2^Department of Stomatology, Chung Shan Medical University Hospital, Taichung 40201, Taiwan; ^3^School of Dentistry, Chung Shan Medical University, Taichung 40201, Taiwan; ^4^Department of Biomedical Engineering, Ming Chuan University, Taoyuan 33348, Taiwan

## Abstract

The penetration depth and the power density of photobiomodulation (PBM) in human tissue under real conditions remain unclear to date. A novel quantitative measurement method was proposed in this study. This study aimed to design a noninvasive measurement system for the quantitative calculation of PBM dose on the attached gingiva. A flexible facial fixture appliance (FFFA) and nine piece detectors were mounted on the retainer to detect the real dose of 660 and 830 nm lasers on the attached gingiva. In addition, the angular distribution of light scattering and the light propagation in the biotissue were obtained. Two cases (a female and a male) are presented in this study. Experimental results demonstrated that the real power density of laser in the target tissue can be measured exactly after the laser light penetrates the orbicularis oris. Simulation results match with real conditions. Conversely, slight differences in power density are observed in the tissue radiated with collimated and uncollimated laser. The proposed method can be used to calculate the real dose in the target tissue for stomatology and deep acupoint stimulation.

## 1. Introduction

Since the first laser device was developed by Maiman in 1960 [1], the effects of laser radiation on the oral tissue have been studied [2, 3]. Mester introduced the use of noninvasive laser radiation as a biostimulator by applying low-level laser therapy (LLLT) to stimulate the biological processes in wound healing [4]. Since then, LLLT has gradually gained popularity from Eastern Europe to the whole world. Nowadays, “photobiomodulation (PBM)” is defined as an accurate and specific term for medical applications [5], and PBM is recommended for noninvasive, painless, and safe biostimulation. PBM can be applied in many clinical symptoms, such as shoulder pain [6], acute neck pain [7], acute low back pain [8], chronic idiopathic orofacial pain [9], temporomandibular joint disorder (TMD) [10], myofacial pain dysfunction syndrome [11], and dentistry [12, 13].

Human skin is the largest human organ, which has a strongly scattering turbid medium. To analyze the photon propagation in biotissue, a previous study punctually radiated the palm of the hand with He-Ne laser and simulated and measured the backscattering light received by a detector in different positions [14]. In addition, two- and four-layer models of the skin were used to simulate light propagation, and the backscattered light received with a detector was published [15]. Recently, the feasibility of performing oximetry at wavelengths of 770 and 830 nm by collecting the backscattered light from two photodetectors at different distances has been studied [16]. However, the backscattered light is insufficient to evaluate the light radiation in the tissue. In addition, transmittance measurement is necessary for PBM.

An in vitro study investigated the impact of different radiation frequency parameters on collagen production in human primary fibroblast cultured in monolayers for optimal PBM [17]. Thus, frequency is a factor that influences collagen production in vitro studies. However, the dose in biotissue is another key factor influencing the therapeutic effects of PBM. Energy density (J/cm^2^) is the dose that the target area receives, and it can be obtained by multiplying power density with time. For PBM therapy, the biphasic response known as “Arndt–Schulz Law” is involved in the optimum dose for tissue wound healing [18]. A recent study has performed the dose analysis of PBM on osteoblasts, osteoclasts, and osteocytes and found that the responses of PBM on these three types of cell differ under different dosages [19]. Thus, dosage is a critical issue for clinical applications, including dentistry. Although PBM has been published by many clinical studies, the penetration depth and the power density of PBM in human tissue under real conditions remain unclear to date. Red laser (600–660 nm) is applied to the skin (epidermis) or shallow acupoints because of the low penetration depth of light, whereas near-infrared (NIR) light is applied to deep tissue or deep acupoints because of its higher penetration depth than red laser. Past works investigated the penetration depth of light in animal model [20] and human “ex vivo” model [21]. Optical fiber probes were also developed for quantitative light dosimetry in tissue [22, 23]. However, the real value of irradiation when the laser penetrates the oral tissue to the gingiva has not been studied. The present study proposes a new method to measure the real power density of light (two wavelengths: 660 nm and 830 nm) after penetrating the orbicularis oris on the attached gingiva. Thus, the healing time of PBM on the target for exact therapeutic dose can be quantitatively calculated for the stomatology, and exact dose of PBM therapy can increase the efficiency of the evidence-based clinical research.

## 2. Materials and Methods

### 2.1. Auto Current Control Circuit Designed for Driving Laser Diode

**Figure 1:**
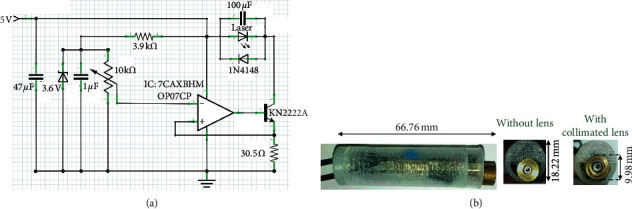
(a) Laser circuit design with auto current control (ACC) and (b) integrated device with and without collimated lens.

The auto current control (ACC) circuit (Figure 1(a)) was designed and realized on a double-sided circuit board (Figure 1(b)). The laser was driven by ACC circuit. One is the aluminum gallium indium phosphide diode laser (model: U-LD-66A051Ap/Dp, Pocket Laser, Union Optronics Corp., Taiwan) at a wavelength of 660 nm ± 5 nm and an output power of 30 mW. The other is the aluminum gallium arsenide diode laser (model: T8350, Pocket Laser, Opto Focus Co., Ltd., Taiwan) with a wavelength of 830 ± 10 nm and output power of 10, 20, and 30 mW. With appropriate warm-up periods, all lasers used in this study had stable output powers.

### 2.2. Detector System Design and Calibration Method

**Figure 2:**
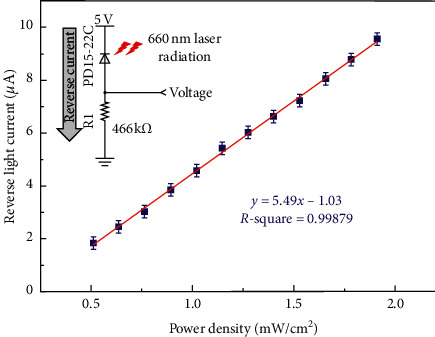
Reverse light current of detector calibration with 660 nm laser radiation.

High speed-sensitive detectors (model: PD15-22C, Silicon PIN Photodiode, Everlight Electronics Co., Ltd., Taiwan) were used in this study. The sensing area of the detector was 2 mm by 2 mm. The reverse light current of the detector radiated by laser (660 nm) was measured in the dark room. The voltage of reverse light current on the resistor (466 k ohm) was obtained with a voltmeter. Thus, the reverse light current of the detector radiated by laser can be calculated by Ohm's law, and the schematic of circuit model measurement is shown in the upper left corner of Figure 2. The relative spectral sensitivity of the detector was 0.96 and 0.74 at 830 and 660 nm, respectively. For instance, the power density of 0.51 mW/cm^2^ corresponded to 0.86 voltage on the resistor by laser. The linear fitting calibration of the detector radiated with laser is shown in Figure 2 (R-square = 0.99879).

### 2.3. Transparent Thin Film

**Figure 3:**
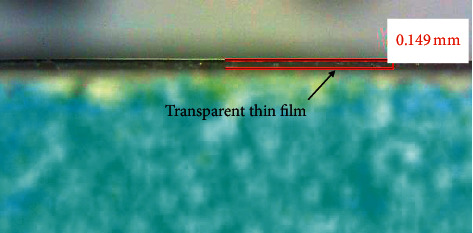
Digital microscope cross-sectional image of transparent PE thin film.

**Figure 4:**
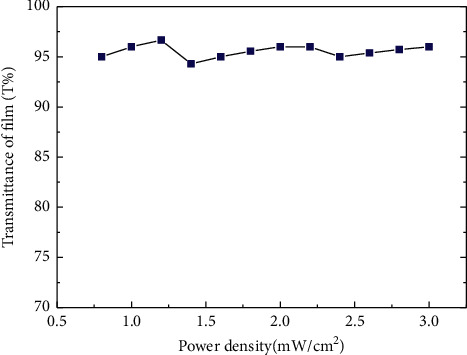
Transmittance of transparent PE thin film.

Nine detector sensing devices were covered by a transparent thin film (polyethylene, PE) to protect the oral tissue. A thin film with a thickness of 0.149 mm was prepared, and its digital microscope cross section (H800X; EMSB) is shown in Figure 3. The transmittance of the transparent PE thin film was measured as 95.55 ± 0.65 (%) by a high-precision power meter (model: EINS OE-Tech Co., Ltd. PM-104) (Figure 4).

### 2.4. Stabilized Flexible Facial Fixture Appliance and Retainer Fabrication

**Figure 5:**
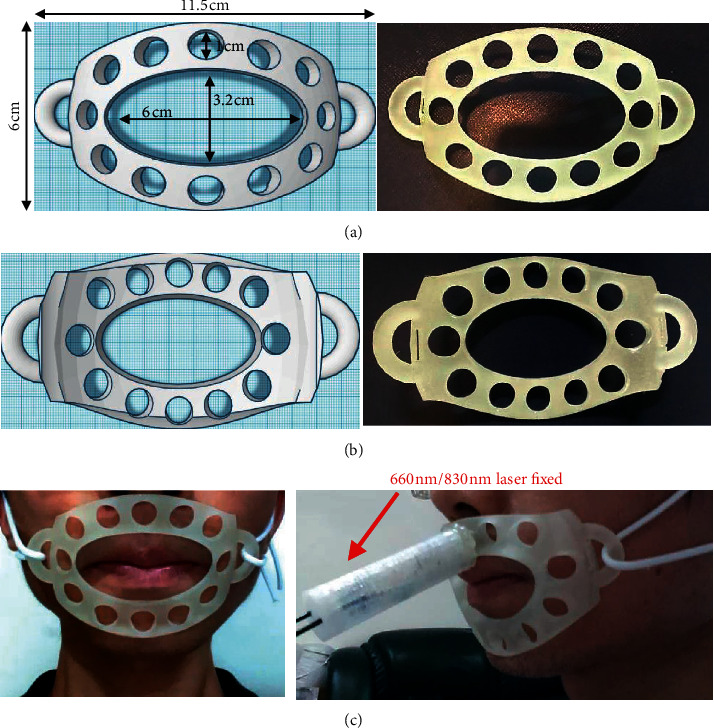
(a) Top view and (b) bottom view of the flexible facial fixture appliance (FFFA). (c) Real experiment situation.

**Figure 6:**
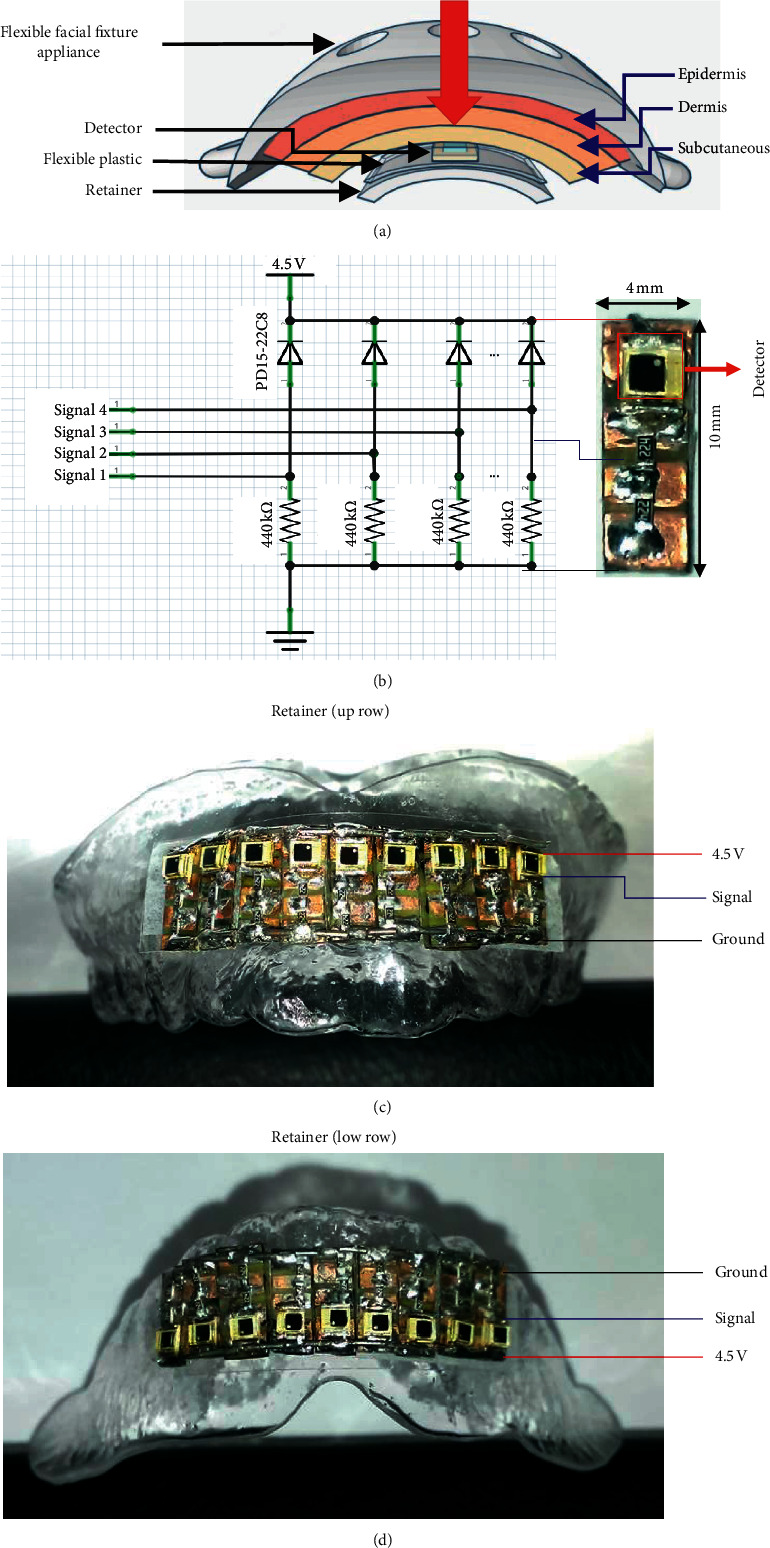
(a) Schematic of the laser power density measured by a detector. (b) Parallel circuit of nine detector devices. (c) Nine piece detectors were mounted on the retainer and worn on the upper attached gingiva. (d) Nine piece detectors were mounted on the retainer and worn on the lower attached gingiva for measurement.

A flexible facial fixture appliance (FFFA) was fabricated by an electro optical systems process (iFaceDesign Technology Inc., Taiwan) that provides a stable position for laser source radiated on the orbicularis oris. The specifications of the FFFA (Figures 5(a) and 5(b)) and the laser radiation application are illustrated (Figure 5(c)). In addition, a retainer (PE terephthalate glycol copolyester, fabricated in the College of Oral Medicine, Chung Shan Medical University) provides a stable mount for the nine detector sensing devices. The schematic of the power density measurement system is shown in Figure 6(a). The sensing circuit design for 4 mm by 10 mm (Figure 6(b)) and sensing devices were mounted on the retainer (Figures 6(c) and 6(d)).

### 2.5. Simulation of Light Scattering and Light Propagation in Skin Layer

For light-tissue interaction, the Henyey–Greenstein phase function *p*(*θ*) was used to describe the angular distribution of light scattering that was influenced by the anisotropy factor as shown below [24]:

(1) $$\begin{array}{c} p\left(\theta \right)=\left(\frac{1}{4\pi }\cdot \frac{1-g^{2}}{{\left(1+g^{2}-2g\cos\left(\theta \right)\right)}^{\left(3/2\right)}}\right), \end{array}$$

where *p*(*θ*) is the scattering probability at angle *θ* and *g* is the anisotropic coefficient. The characteristic of anisotropy factor *g* gives absolute values from 0 to 1 (*g* = 0 shows an isotropic scattering, *g* = 1 represents forward scattering, and a negative value stands for backward scattering [25]). The different angular distributions of light scattering at wavelengths of 660 and 830 nm were simulated in this study.

The source function model was proposed on the basis of the light absorption and scattering in the tissue [26]. The light intensity propagation at the (*r*, *z*) point in the biomedia, termed I (*r*, *z*), was calculated as follows [27]:

(2) $$\begin{array}{c} I\left(r,z\right)=I_{o}e^{-\left(\mu_{a}+\mu_{s}\right)z}e^{\left[-1/2{\left(r/\sigma \left(z\right)\right)}^{2}\right]},\\ \sigma \left(z\right)=\sigma_{o}e^{\left(\mu_{s}z|/|2\right)}, \end{array}$$

where *μ*_a_ is the absorption coefficient, *μ*_*s*_ is the scattering coefficients, *σ*_*0*_ is the standard deviation of beam, *σ*(*z*) represents the characteristic radius for the geometric cross section of the light beam at depth *z*, and *r* is the light intensity in various depths of radial distance. In this study, the phase function *p*(*θ*) and the laser intensity propagation were simulated with red and NIR laser in the skin layer by MATLAB 2015b. In a male, the thickness of the orbicularis oris is 0.98 cm and the simulated condition is 1.33 cm. The angular dependence of light scattering and the light propagation were also predicted in the different skin layers.

## 3. Results

**Figure 7:**
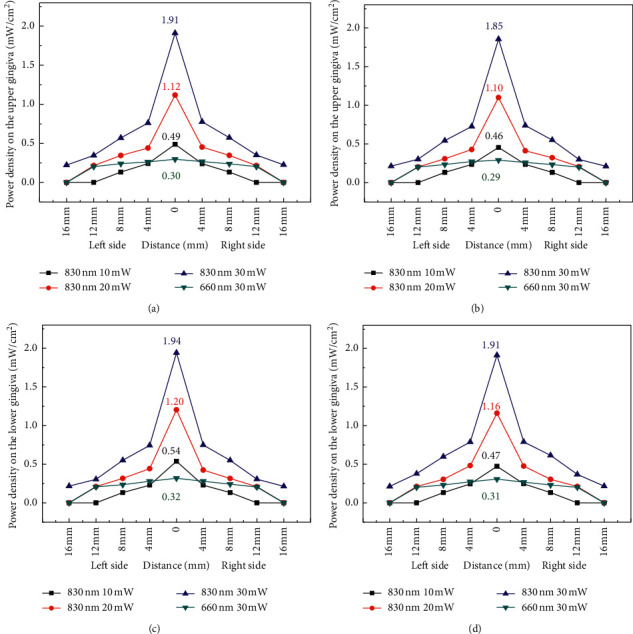
Power density analysis for (a) collimated and (b) uncollimated laser on the upper attached gingiva for a male. Power density analysis for (c) collimated and (d) uncollimated laser on the lower attached gingiva for a male.

**Figure 8:**
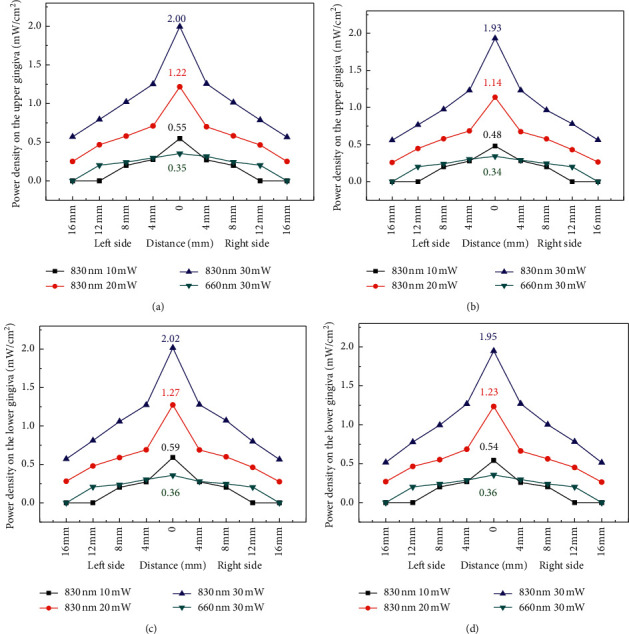
Power density analysis for (a) collimated and (b) uncollimated laser on the upper attached gingiva for a female. Power density analysis for (c) collimated and (d) uncollimated laser on the lower attached gingiva for a female.

The power dependence of laser at 660 and 830 nm on the upper attached gingiva (Figures 7(a) and 7(b)) and lower attached gingiva (Figures 7(c) and 7(d)) was analyzed in a male. In the same way, the power densities on the upper attached gingiva (Figures 8(a) and 8(b)) and lower attached gingiva (Figures 8(c) and 8(d)) were analyzed in a female. Laser at 830 nm can penetrate tissue deeper than laser at 660 nm. Thus, the power density of laser at 830 nm is approximately six times greater than that of laser at 660 nm (Figures 7(a)–7(d) and 8(a)–8(d)).

**Figure 9:**
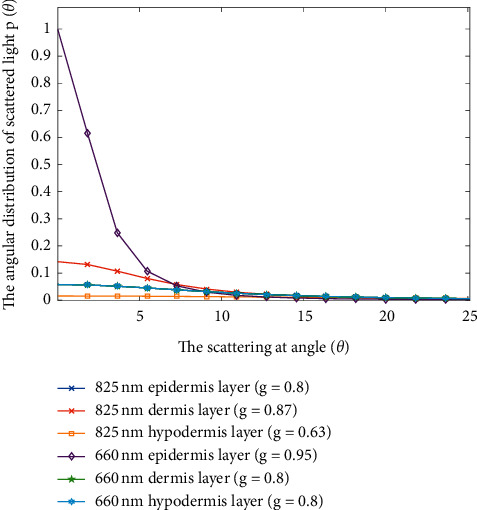
Angular distribution versus light scattering angle.

The anisotropic coefficient (*g*) of angular distribution was used to simulate the light scattering at 660 nm [28, 29] and 825 nm [30, 31]. The wavelength of 660 nm indicated the most scattering in the epidermis layer of angular distribution by light scattering (Figure 9). On the basis of the experimental results, the uniform distribution of power density on the attached gingiva by laser (660 nm) can be considered a large proportion of light scattering in the epidermis layer by the phase function simulation.

**Figure 10:**
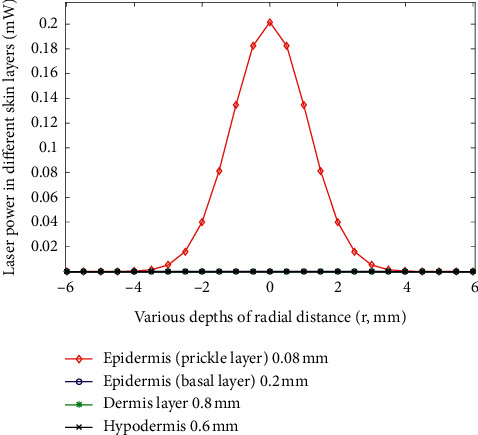
Laser power at wavelength of 660 nm in different skin layers.

**Figure 11:**
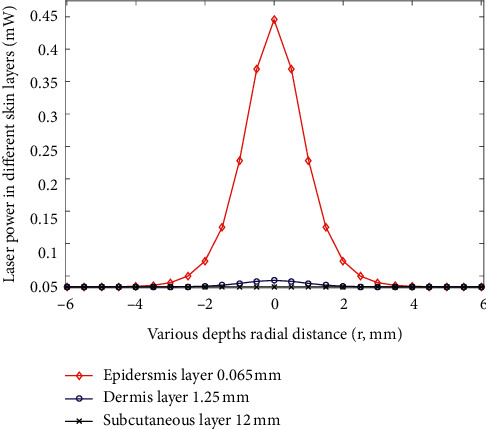
Laser power at wavelength of 825 nm in different skin layers.

The simulation results revealed stronger light intensity propagation in the epidermis layer for the 660 nm laser (input power set at 1 mW for simulation) than the 825 nm laser (Figure 10). The optical parameters of *μ*_a_ and *μ*_s_ as mentioned in previous studies were used [28, 29]. However, the light intensity propagation in the dermis layer was stronger with the 825 m laser than the 660 nm laser (optical parameters of *μ*_a_ and *μ*_s_ as mentioned in previous studies were used [30, 31]). This result indicates that the NIR light provided higher penetration depth than the red light (Figure 11). The corresponding radial spread can be considered the occurrence of multiple scattering in the skin. Furthermore, the power densities on the attached gingiva measured with two wavelengths of radiation for a male and a female were compared in this study (Tables 1 and 2).

## 4. Discussion

Light wavelength influences the deep and superficial target tissues at long and short wavelengths, respectively [32]. The efficacy of the 810 nm laser is significantly higher than that of the 660 nm laser in treating trigeminal neuralgia and TMD [9]. The action spectral wavelength ranges from 600 nm to 850 nm for cellular responses, but the inactive region is from 700 nm to 750 nm [33]. The wavelengths of 660 nm (for superficial target) and 830 nm (for deep tissue) used in this study are located in this therapeutic window. The effects of PBM in this therapeutic window are attributed to the action and absorption spectra of cytochrome c oxidase activity and adenosine triphosphate synthesized content [34]. The penetration depth increases with increasing wavelength of light [35]. Thus, the suitable wavelength is an important factor for superficial target and deep tissue (or acupoints).

Another important factor is the dose dependence of PBM for cellular action [19]. In the present study, light dose analysis was performed to measure the power density of light after penetrating the orbicularis oris. On the basis of the experimental results (Figure 8(a)), the highest power density (1.94 mW) on the central area of the attached gingiva can be detected when the collimated laser (830 nm) radiates on the orbicularis oris. The small difference in power density on the attached gingiva between the collimated and uncollimated laser was obtained (Figures 7 and 8). Then, the power density decayed quickly along two sides until 16 mm distance. Simulation results (Figure 11) showed that the peak power of NIR light (825 nm) propagation in the subcutaneous layer can be obtained as 0.033 mW when the input power is 1 mW. Thus, the peak power can be estimated to be approximately 0.99 mW when the incident power of laser was 30 mW for simulation. The simulation results can help understand the light propagation of laser (830 nm) in tissue. On the basis of the experimental results, the power density of laser (660 nm) indicated a broadband distribution (Figures 7 and 8) on the upper and lower attached gingiva. Similarly, the laser wavelength at 660 nm indicated that a larger proportion of scattering occurred on the epidermis layer by the phase function compared with the 825 nm wavelength (Figure 9). Thus, a broadband distribution of power density (660 nm laser) can be considered the multiple scattering in the epidermis layer leads to a lower transmittance in the orbicularis oris. By contrast, the collimated laser (830 nm) provided a higher power density on the attached gingiva compared with the 660 nm laser. After around 16 mm from the laser output center, the power density decreased to approximately zero on the attached gingiva at 660 nm wavelength. Furthermore, the power densities at the two wavelengths between a male and a female were compared in this study (Tables 1 and 2). The gingiva of the female indicated a higher power density after laser (830 nm) radiation because of the thinner orbicularis oris and less beard than the male. The orbicularis oris muscles in the upper and lower attached gingiva were 9.80 and 8.72 mm thin for the male, respectively, and 7.72 and 6.88 mm for the female, respectively.

On the basis of the adequate radiation of PBM in biological tissues, the energy density (*E/a*)_act_ of PBM including the beam cross section *a*, the intensity of stimulation *I*_stim_, and the total irradiation time ∆*t*_tot_ can be expressed as follows [36]:

(3) $$\begin{array}{c} {\left(\frac{E}{a}\right)}_{\text{act}}=I_{\text{stim }}\Delta t_{\text{tot }}. \end{array}$$

The effective energy density range is given by the particular Arndt–Schultz curve. According to Arndt–Schulz law, low-energy density stimulation excites physiologic activity, moderate energy can activate cellular functions, and high energy densities inhibit physiological activity for the dose-dependent relationship and biologic response [18, 37]. Low-intensity light dose (1 J/cm^2^) enhances osteoblast proliferation, osteoclast differentiation, and osteoclastic bone resorption activity, but osteocyte proliferation is not observed even at high doses (5 J/cm^2^) in vitro [19]. The cell activities indicate that dose dependence is a critical factor for PBM. Experimental results showed that the dose reaching the target area decreased tremendously in deep tissue and changed with depth. This result explains the different outcomes of photobiomodulation therapy, considering that most clinical trials of PBM did not define the real dose of the target tissue, especially deeper tissues.

**Figure 12:**
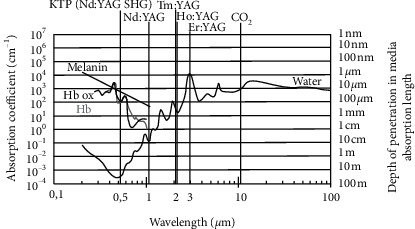
Absorption spectrum of hemoglobin (Hb), hemoglobin-originated (Hb ox), melanin, and water with several lasers. Reprinted with permission from H.-O. Teichmann [41].

For laser beam radiation on the skin, reflectance accounts for 3% when the laser is directed toward tissue [38]. About 93%–96% of the incident radiation not returned by regular reflectance could be absorbed and scattered [39]. The epidermis is the outermost portion of the skin, and the layer is approximately 75–150 *μ*m in thickness [40]. The living epidermis contains melanosomes and melanin, which are the principal light absorber factor of this layer. The absorption coefficient shows an inverse proportion in melanin from the ultraviolet to NIR light spectrum (Figure 12) [41]. The scattering of the epidermal layers facilitates the strong forward scattering for the epidermis layer and the stratum corneum layer [42]. The dermis is the second layer, and the layer of fibroelastic connective tissue is 1–4 mm thick [40]. Dermis is made of two connective tissue layers: papillary and reticular layers. The papillary layer contains more loosely distributed elastic and collagen fibrils than the underlying reticular layer [43]. By contrast, the reticular layer contains dense collagenous and elastic connective tissues, which constitute the greater bulk of the dermis [44]. For dermis connective tissue layers, scattering in tissue is dominated by the reticular dermis, and the main absorbers in the dermis are blood hemoglobin in oxygen saturated (oxy), desaturated forms (deoxy), carotene, bilirubin, and water [45]. The hypodermis is the third major skin layer composed of loose fatty connective tissue, which causes light scattering by spherical droplets of lipids. The absorption in the hypodermis is determined by hemoglobin, lipids, and water [43, 45].

Laser (660 nm) with 4 J/cm^2^ is better than 20 J/cm^2^ in accelerating the oral wound healing of rats [46]. PBM is an effective method to promote healing following gingivectomy for patients by laser (685 nm) with 4 J/cm^2^ [47]. An attenuation of light intensity along the frontal and occlusal views is 50% at a 3 mm distance from the laser probe (660 nm) [48]. The present study proposes a quantitative dose analysis method for the light penetrating the orbicularis oris on the attached gingiva. The power density distribution by laser (660 nm and 830 nm) irradiation was investigated, and results showed that the 830 nm laser was more suitable than the 660 nm laser for deeper biotissue stimulation. Furthermore, PBM can be operated easily from the orbicularis oris with FFFA. Our study shows that the power density at 830 nm wavelength on the attached gingiva for treatment can be quantitatively analyzed, and the result has a great potential for dental applications. However, the only limit to the proposed experimental model is the thickness of the orbicularis oris. It means we cannot measure the real dose in deeper tissue than orbicularis oris.

## 5. Conclusions

The quantitative dose analysis of PBM on the attached gingiva tissue has been proposed and demonstrated. That means the dose under the skin can be clearly defined and calculated for PBM therapy. The real power density on the attached gingiva can be reached by radiating NIR laser light from outside. We believe the dentists and the patients will be benefited with NIR PBM therapy. Besides, exact dose calculation will help promote the efficiency of the evidence-based clinical research.

## Figures and Tables

**Table 1:** Power density of the upper attached gingiva for a male and a female with two lasers.

Upper attached gingiva measurement (male (age: 30), left/female (age: 28), right)				
Laser (nm)	Power (mW)	Position	With lens	Without lens
			Power density (mW/cm^2^)	Power density (mW/cm^2^)
830	10	Center	0.49/0.55	0.46/0.48
	20	Center	1.12/1.22	1.10/1.14
	30	Center	1.91/2.00	1.85/1.93
660	30	Center	0.30/0.35	0.29/0.34

**Table 2:** Power density of the lower attached gingiva for a male and a female with two lasers.

Lower attached gingiva measurement (male (age: 30), left/female (age: 28), right)				
Laser (nm)	Power (mW)	Position	With lens	Without lens
			Power density (mW/cm^2^)	Power density (mW/cm^2^)
830	10	Center	0.54/0.59	0.47/0.54
	20	Center	1.20/1.27	1.16/1.23
	30	Center	1.94/2.02	1.91/1.95
660	30	Center	0.32/0.36	0.31/0.36

## Data Availability

The data used to support the findings of this study are included within the article.
